# Elevating the role and professional contributions of the neonatal dietitian for the future: insights from the experts themselves

**DOI:** 10.3389/fped.2025.1639283

**Published:** 2025-08-26

**Authors:** Deborah M. Abel, Alayne M. Gatto, Jennifer O. Fowler, Christina J. Valentine

**Affiliations:** ^1^ Dietetics and Nutrition, Florida International University, Miami, FL, United States; ^2^ East Carolina University Health Medical Center, Greenville, NC, United States; ^3^ Banner—University Medical Center Tucson, Tucson, AZ, United States

**Keywords:** neonatal dietitian nutritionist, survey, hospital administration, NICU (neonatal intensive care unit), career advancement

## Abstract

**Background:**

The neonatal registered dietitian nutritionist plays a crucial role in the care of premature and critically ill infants in the neonatal intensive care unit. Critical care success with such vulnerable patients requires expertise in patient-centered care, multidisciplinary collaboration, and adaptive clinical problem-solving. This research aimed to identify the needs, engagement levels, and expertise of the neonatal registered dietitian nutritionist while also providing insights into their job satisfaction and career longevity.

**Methods:**

This was a cross-sectional examination using a national, online, IRB-approved survey completed by current and former neonatal practicing dietitians. In addition to descriptive statistics, the Chi-Squared test and Fisher's Exact test were used for categorical statistical analysis.

**Results:**

253 current (*n* = 206) and former (*n* = 47) participants completed the online questionnaire. Before role as a neonatal registered dietitian nutritionist, 210 respondents, 84 (40%) reported having pediatric clinical experience, 94 (44%) had clinical pediatric dietetic intern experience, 21 (10%) had previously worked as a community-based pediatric nutritionist, 15 (7.1%) had specialized pediatric certification or fellowship, and 12 (5.7%) had no prior experience. Of 163 respondents, 83 (50.9%) reported receiving financial support or reimbursement for additional neonatal training. Respondents who felt valued as team members planned to stay in the neonatal registered dietitian nutritionist role for more than 5 years (*p* > 0.0046). Additionally, they reported having acknowledgement and appreciation (64.4%), motivation (54.1%), and opportunities for advancement (22.9%).

**Conclusion:**

Neonatal registered dietitian nutritionists do not have a clear competency roadmap, nor a defined career development track. In addition, financial support or reimbursement for continuing education is not consistently an employee benefit, which may play a key role in job satisfaction and retention. This data provides valuable insights for not only managers of dietitians but also professional societies and hospital administration to build career and employee retention opportunities, and to ensure safe patient care for the smallest hospital patients.

**Clinical Trial Registration:**

ClinicalTrials.gov, identifier NCT06771778.

## 1 Introduction

In the United States, more than 1,400 hospitals have been identified as having advanced care of neonates in Level 2, 3, or 4 nurseries (1). To ensure that every infant in a Neonatal Intensive Care Unit (NICU) receives standardized care with optimal utilization of resources and medical personnel, the American Academy of Pediatrics developed the Standards for Levels of Neonatal Care. This family-centered care implementation tool identifies the minimum staffing requirements for each level of neonatal care (2).

The role of the neonatal registered dietitian nutritionist (RDN) is to implement evidence-based, individualized care for the growth and neurodevelopment of infants, both in the short and long term (3). Although the extent of the time devoted to the role of the NICU RDN may be variable due to the acuity of the NICU, the facility should have at least one RDN identified with specialized training in neonatal nutrition (2). The specialized training of the RDN should include the practices of value-based care, considerations for quality improvement, and the safety of the vulnerable preterm infants (4, 5). The RDNs are the experts who provide recommendations for the nutritional care of the infant. They may participate in daily or weekly medical rounds and in a multi-disciplinary approach, potentially including a speech therapist, physical therapist, pharmacist, bedside nurse, nurse practitioner, and neonatologist. The NICU RDN will review the daily and weekly growth status, make nutritional recommendations, and address social needs impacting infant nutrition (6). Furthermore, the careful, daily monitoring of nutrition by the RDN assists the team in financial stewardship and patient risk management. NICUs with stronger teamwork and an increased safety climate experience a lower rate of healthcare-associated infections (7).

Not all US hospitals have adopted the utilization of the neonatal RDN even with strong evidence that they significantly improve growth outcomes (measurements of weight, length, and head circumference) (8) and promote cost containment (9). Additionally, the NICU may not be adequately RDN staffed when considering the level of acuity and number of babies that need to be followed, although specific requirements for staffing of the RDN in hospital NICUs have not been validated across the profession (10). However, their value has been substantiated, and the hospital should take ownership to ensure there are opportunities for advancement, appropriate compensation, and job security to keep the NICU RDN in the role and avoid a cyclical vacancy. Furthermore, exploring burnout involves comparing full-time equivalent (FTE) employees to the number of beds (20) and length of employment, as these factors may affect employee turnover.

The neonatal RDN plays a crucial role in the care of premature and critically ill infants in the NICU. Additional skill sets may not be taught in their nutrition degree coursework or dietetic internship. Dietetic internships do not require a pediatric rotation, and only a few pediatric-focused internships currently exist. The Accreditation Council for Education in Nutrition and Dietetics (ACEND) updated in 2024 to require a master’s degree to become a credentialed RDN (11); however, advanced-level courses in neonatal nutrition, including classes and fellowships that provide unique expertise and skills for the NICU, are limited. Only a few dietetic internships have pediatric rotations, while others may not have any NICU or pediatric coursework (12). With added costs and schedule constraints, it may be challenging to participate in specialized neonatal nutrition fellowships, conferences, or graduate pediatric certificates. However, once the RDN obtains NICU job experience, the Commission on Dietetic Registration offers an exam for specialty credentials to become board-certified in pediatric nutrition (CSP) and/or pediatric critical care (CSPCC) after the RDN has a minimum of 2,000 hours in the specialty, highlighting advanced skillsets (11).

To be successful in this specialized nutrition field, beyond medical nutrition therapy, RDNs should possess personal skills and qualities that include effective communication with the team, compassion, cultural humility, time management, and emotional resilience (13). During and even after the COVID-19 pandemic, hospital healthcare systems suffered a massive loss of employees and decreased employee morale (14, 15). Entry-level RDNs with little experience and without opportunities for job shadowing were hired into roles without expertise and specialized training. NICU RDNs are also frequently assigned to cover other hospital areas where staffing is not adequate or on weekends/off-hours, without additional incentives.

In recent publications on the role of the NICU RDN, the authors recommended more structural support for neonatal RDN, including tools, staffing, and resources for continuing professional development and practicing excellence (16, 17). Literature related to the requirements—skills, specialized education/training, competencies—for entry-level RDN is limited. Continuing education is required to maintain ongoing competency and job satisfaction. Physicians and other healthcare team providers will trust and respect the NICU's RDN when they have the appropriate expertise and competence. Neonatal RDNs need to earn and build collaborative, trusting relationships with the physicians in the unit they serve, and it may be even more complicated as there are often multiple neonatal health care providers (18). Building these relationships may be integral for the neonatal RDN to remain personally motivated and vested in a team and feel intrinsically motivated to stay in the role.

A survey was designed to determine the needs of the NICU RDNs. It included what will keep the NICU RDN engaged, continue to learn, and passionate about their expertise (workplace motivation, education, mentoring). It reported insight into job satisfaction and longevity in a career choice as a neonatal dietitian. In addition, the survey provided data and feedback from the NICU RDNs that can potentially improve their specialized education and training and, ultimately, improve the safety and nutritional care of the neonate. Lastly, the survey provides evidence to organizations and hospitals that support NICU RDNs to reevaluate the value of the role, appropriate compensation for skillsets, and potential career opportunities for growth and development. This study utilized survey questions to determine whether neonatal dietitians have obtained adequate, specialized education and training to result in outcomes such as job satisfaction, longevity in role, and personal growth. These factors, when combined, will ultimately foster a stronger commitment to achieving optimal outcomes for their patients, and together, form a more cohesive unit of NICU practitioners.

## 2 Materials and methodology

### 2.1 Study design and population

A Qualtrics survey enabled a cross-sectional design to reflect the perspectives of Neonatal and Pediatric Registered Dietitian Nutritionists (RDNs) currently or formerly, within the last 20 years, working in Neonatal Intensive Care Units (NICUs) in the neonatal dietitian role and ensuring a comprehensive understanding of the role across different stages of employment. The survey addressed skilled competencies, training and education, and challenges to job satisfaction. This comprehensive survey came to fruition through neonatal dietitians expressing current job concerns and frustrations in a neonatal RDN forum, and subsequently, was initiated by the authors developing questions and reviewing prior survey publications in the literature. The survey was validated and pilot-tested with a small test group of RDNs prior to administration to ensure feasibility and minimize potential survey fatigue. The national IRB-approved online survey was distributed through established dietitian network channels, including online listservs and communities within Neonatal and Pediatric Dietitian practice groups and organizations, targeting current and former NICU RDNs within the last 20 years. Participants received the online Qualtrics survey via a unique link or QR code to complete. The survey was designed to take approximately 10 min with 23 questions, ensuring that the time commitment would not be a burden for participants to complete.

Participation in the survey was voluntary, and all participants were informed of their rights, including the confidentiality of their responses and the fact that they could withdraw from the study at any time with a copy of the consent form provided. To further encourage participation, participants were offered an optional incentive: a small monetary $10.00 e-gift card, which would be sent upon survey completion via email or text via their provided contact information. This information was also requested to avoid potential duplication of response. This optional gift card incentive, limited by budget allocation, was offered to the first 250 participants who completed the survey, and the link remained open until the gift card allocation was exhausted. The survey opened for approximately one week from March 25th to April 2nd, 2024, during which 260 surveys were submitted. The survey results were stored in a password-protected online site via Qualtrics.

### 2.2 Survey design

Supplementary Materials (Supplementary Table S1) show the survey instrument that consisted of two main parts.

#### 2.2.1 Part I: employment and training details

Questions in this section focused on respondents' current or former employment as a Neonatal RDN, the duration of their NICU employment, and the classification and size of the NICU in which they worked. Additionally, questions were included about the institutional support for NICU training, how respondents obtained continuing education, and whether they had used ChatGPT for education. Questions such as “Are you currently employed as a Neonatal Registered Dietitian Nutritionist?” and follow-up questions (e.g., “If no, how long ago were you employed as a Neonatal RDN?”) helped segment participants. They directed them to appropriate sections of the survey. The questions in Part 1 were designed to capture the scope of experience in the NICU setting and explore reasons for career transitions. The survey design was adaptive based on the responses and allowed respondents to bypass irrelevant questions (for example, no longer practicing) and move to the next section; however, all questions were required to be answered to advance to the next question to obtain complete surveys and receive the gift card.

#### 2.2.2 Part II: experience and career development

This section inquired about respondents' previous clinical experience before becoming a NICU RDN, their onboarding and training, their feelings of being a valued member of the NICU team, additional credentials held, recognition within the NICU setting, job responsibilities, and career plans. These questions were designed to assess professional development opportunities and the role of ongoing education in shaping NICU dietetics practice.

### 2.3 Statistical analyses

The data collected through the survey were analyzed using Stats IQ, a statistical tool integrated within the Qualtrics platform. Frequencies and percentages were calculated for categorical variables such as employment status, NICU classification, and continuing education methods. Respondents were allowed to select all applicable options for questions requiring multiple answers (e.g., reasons for leaving a role or methods for obtaining continuing education). No reported survey data was excluded. Descriptive data were presented as percentages to illustrate trends and patterns in NICU RDN employment, training, and career development. As a purely cross-sectional survey, there were few planned comparisons; thus, statistical corrections are not necessary.

To analyze differences in categorical variables of this exploratory research, the Chi-Squared test and Fisher's Exact test were utilized. These tests were selected based on the nature of the data and the need to assess whether observed differences in response distributions were statistically significant. Standard deviations and means were utilized in normally distributed data, and medians were employed for non-normally distributed data. A statistically significant level of *p* < 0.05 was used in all analyses of comparison data.

The Chi-Squared test was used to examine larger sample sizes with expected frequencies greater than five, while Fisher's Exact test was used for smaller sample sizes or when expected frequencies were below five. Both tests provided valuable insights into potential relationships between demographic variables (such as years of experience, professional background, and practice settings) and various aspects of dietetic practice in NICUs.

## 3 Results

### 3.1 Demographics and employment information

Two hundred and sixty respondents participated in the survey, with 253 (97.3%) indicating current or former employment as a Neonatal RDN. Of these, 206 (81.4%) were currently employed as Neonatal RDNs, while 47 (18.6%) had been employed in the past. Most respondents (73.9%) reported working full-time at the NICU, with the remaining 26.1% working part-time. Among part-time workers, 40.9% spent most of their hours in adult care settings, while 36.4% worked in pediatric inpatient or outpatient care.

Regarding NICU classification, 54.7% of respondents worked in Level 3 NICUs, 41.9% worked in Level 4 NICUs, and only 3.5% were employed in Level 2 NICUs. When asked about NICU bed capacity, most respondents, 48.8%, reported working in units with 21–50 beds, and 36% worked in units with 51–99 beds. There is no statistically significant relationship between “Does your NICU use the 20 NICU beds per Full Time Equivalent (FTE) RDN for staffing to screen and assess the infants in the unit” and “How long were you employed as a NICU RDN?” (*p* = 0.6877).

### 3.2 Institutional support and continuing education

Regarding institutional support for NICU training, most respondents (77.3%) reported receiving education hours without needing to utilize vacation hours. In comparison, 60.7% received financial support or reimbursement for training, and 50.9% received support for conference attendance. Additionally, 35% had the opportunity to shadow another NICU RDN as part of their professional development.

For ongoing education, the most common methods for obtaining NICU-related continuing education were webinars (95.9%), live or virtual conferences (84.9%), self-study materials (71.5%), and industry-supported events (71.5%). Only a small proportion (23.3%) participated in Academy of Nutrition and Dietetics-sponsored events. A few respondents (1.7%) reported using ChatGPT for their education or learning.

### 3.3 Workplace and role experience

The survey also explored respondents' work experience before becoming a NICU RDN, as represented by Table 1. Most (62.4%) had adult clinical experience, followed by 44.8% with pediatric dietetic intern clinical rotation experience. Notably, only 7.1% of respondents had specialized pediatric certificates or fellowships before working in the NICU.

**Table 1:** Survey part II: experience .

When you started as a NICU RD, what experience did you have? (Check all that apply)		
	*N* = 210	Percent %
Adult experience	131	62.4%
Pediatric dietetic intern clinical rotation	94	44.8%
Pediatric Clinical Experience	84	40.0%
WIC Nutritionist	21	10.0%
Other	18	8.6%
Specialized pediatric certificate or fellowship	15	7.1%
None	12	5.7%

When starting their roles, most respondents (71.9%) received onboard training from a tenured NICU RDN, as highlighted in Table 2. The most common additional credentials held by NICU RDNs included a master's degree (72.6%), and certification in nutrition support (CNSC), 42.5%. In 2024, a master's degree became required for eligibility to take the registered dietitian exam; however, tenured dietitians are exempt (9).

**Table 2:** Survey part II: experience.

Did you have onboarding training from a tenured NICU RD?		
	*N* = 210	Percent %
Yes	151	71.9%
No	59	28.1%

### 3.4 Recognition and career development

A significant portion of respondents reported being recognized in their roles. The most common forms of recognition included acknowledgment and engagement in NICU rounds (94.2%) and being regularly consulted by the NICU team for expertise (87%). Furthermore, most respondents (92.8%) reported feeling like valued members of the NICU team. Additionally, 64.3% of respondents had been thanked by a family member for caring for their infant patients. However, as reported in Table 3, less than a quarter (20.8%) felt appreciated by the administration outside their yearly review.

**Table 3:** Recognition.

How are (were) you recognized in your NICU RD position? (Check all that apply)		
	*N* = 207	Percent %
Acknowledgement and Engagement in NICU rounds	195	94.2%
Consulted regularly by the NICU team for your expertise	180	87.0%
Noticed by the NICU team when you were absent.	167	80.7%
Thanked by a family member for the care of their baby	133	64.3%
Administration Appreciation (outside of your yearly review)	43	20.8%
Acknowledgement and Engagement in NICU rounds	195	94.2%

**Figure 1:**
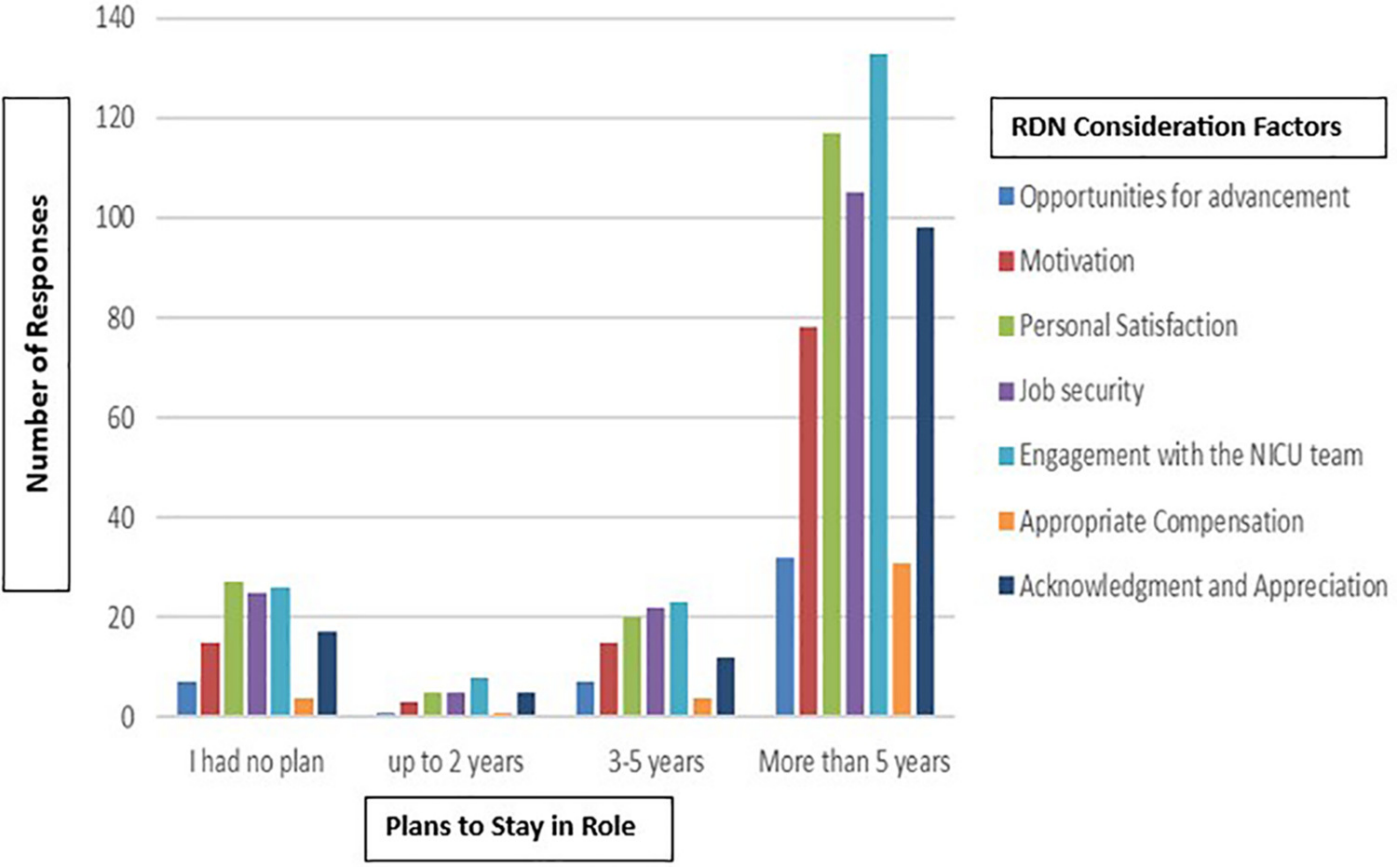
Comparison of questions: “Do/Did you feel you have the following in your role in the NICU…” and “Do/Did you plan to stay in your role for …?”.

When asked about career satisfaction, as displayed in Figure 1, most respondents reported having personal satisfaction (82.4%), job security (76.6%), and engagement with the NICU team (92.7%). However, fewer respondents felt they had opportunities for advancement (22.9%) or appropriate compensation (19.5%). A large proportion (65.4%) of respondents indicated they planned to remain in their current NICU RDN role for more than five years, while 18.3% had no plan to stay. Respondents who felt valued as team members planned to stay in the NICU RD role for more than 5 years (*p* < 0.0046). Additionally, they reported having acknowledgement and appreciation (64.4%), motivation (54.1%), and opportunities for advancement (22.9%).

### 3.5 Job responsibilities

Job responsibilities varied, with most NICU RDNs reporting that they precepted dietetic interns (86.7%) and taught residents (53.6%). Fewer respondents were involved in research (32.7%), NICU follow-up clinics (21.9%), or management roles (15.8%). The survey also assessed how often RDNs' clinical notes were read by other healthcare providers. Approximately 52.4% reported that their notes were consistently read, while 34.8% said they were sometimes read. Barriers to note review included cumbersome electronic medical records (21.4%) and lack of interest from providers (17.3%).

## 4 Discussion

**Figure 2:**
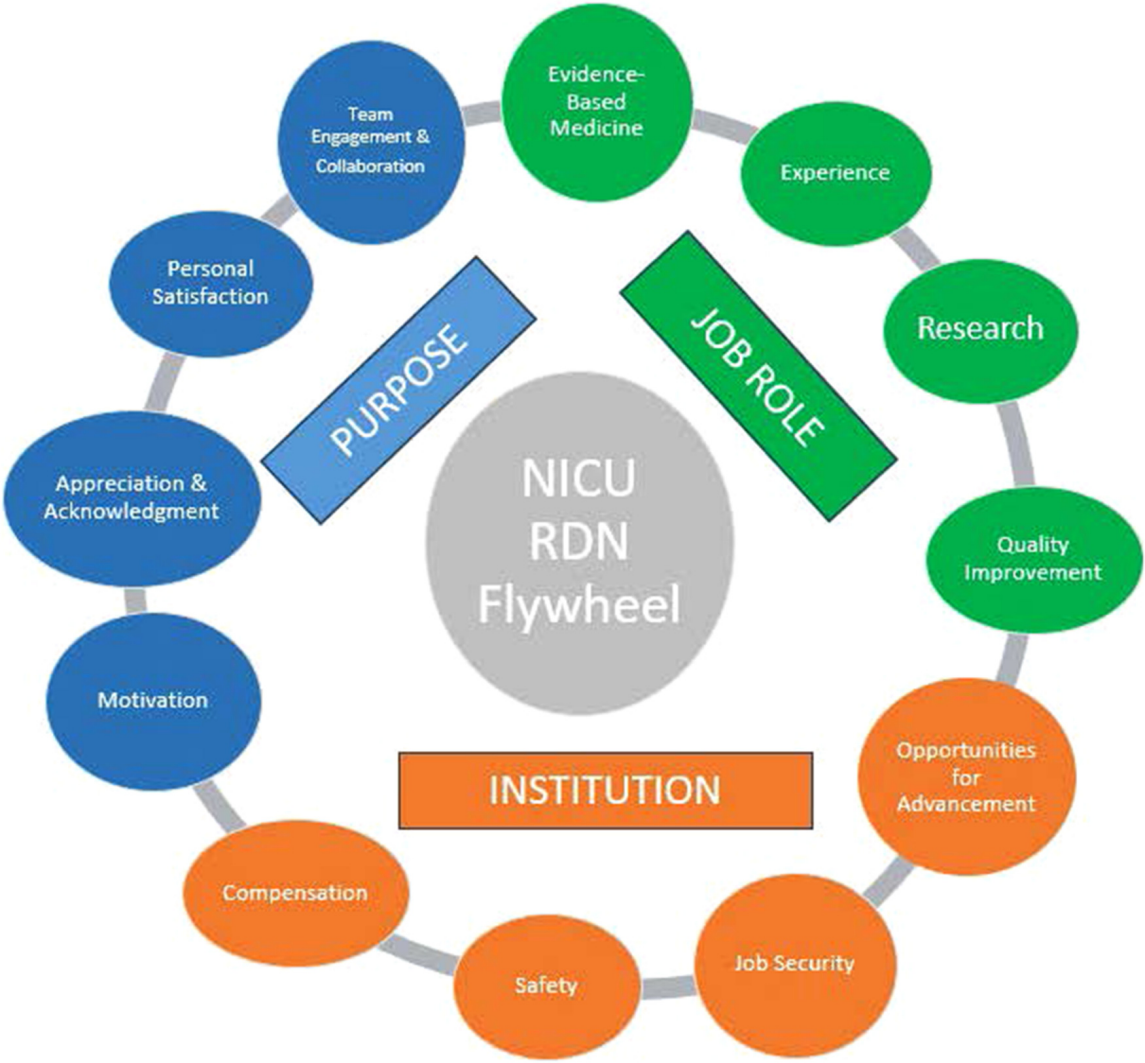
NICU RDN flywheel.

NICU RDNs as a specialty do not have a unified competency roadmap, career development track, or designated team role, however, as indicated in Figure 2, the survey has identified many roles and traits of the neonatal RDN to designate their purpose, job role, and needs from the hospital institution. This survey provided insights into several shortcomings that hinder the acquisition of such expertise, including limited formal educational training for pediatric RDNs. These limitations can be addressed through academically rigorous and pediatric rotations during their dietetic internship. Respondents provided insights into previous work experience, as the majority were trained as adult RDNs and pursued pediatrics through personal desire, professional development, or career availability. The specialty of neonatal nutrition requires additional skills to become proficient; however, it was reported that dedicated training is lacking for most before entering this specialized area of nutrition, and very few respondents responded that they had been formally trained or had specialized pediatric certificates or fellowships before working in the NICU. Seeking training from a fellow tenured RDN is valuable, suggesting that this type of training should be considered as a standard for onboarding new dietitians, as it is cost-effective, feasible, and does not require financial support to advance training.

The neonatal RDNs reported that most training resulted from continuing education, since, as for many with limited education funds, relying on low or no-cost offerings may be helpful. The most common methods for obtaining NICU-related continuing education were webinars, live or virtual conferences, self-study materials, and industry-supported events. Only a small proportion participated in Academy of Nutrition and Dietetics-sponsored events, which may reflect the cost of membership or activities. Continuing education may be limited to specific topics and lack curriculum criteria for broader NICU-specific learning experiences and testing knowledge levels.

### 4.1 Summary recommendations

**Demographics and Employment Information—**A standard for the patient-to-RDN ratio in the NICU is warranted. Further investigation is needed to determine the impact of the suggested RDN staffing on safety and efficiency, while also avoiding burnout.

**Institutional Support and Continuing Education**—Part of the employee benefits should include budgeted and hospital-paid neonatal specialized training and continuing education. Hospital administration needs to recognize the importance of further education to obtain the expertise required for the role of the NICU RDN.

**Workplace and Role Experience**—Dietitians need increased access to additional specialized NICU training during their internship or college education. Accrediting bodies of the dietetic professional organization should prioritize and focus on the chosen career pathway of the future RDN.

**Recognition and Career Development**—Hospital administration and NICU staff need to become more knowledgeable about the importance of the NICU RDN as a highly valued NICU team member. A follow-up longitudinal study of the role, efficiency, and cost-effectiveness of the NICU RDN should be considered.

**Job Responsibilities**—Clinical Nutrition Managers and the neonatal unit need to explore the most effective way of utilizing and documenting NICU RDN recommendations into patient care. Time should be considered for more value-added activities, including more direct patient/caregiver engagement, teaching pediatric medical residents, precepting future RDNs, and in research.

Strengths of this study included a robust and expedited survey response. Limitations in this study include the inability to have complete random sampling, as neonatal dietitians were sought out using listservs, community groups, organizational distribution lists, and personal/professional contacts. Although geographical and institutional diversity was not known, each dietitian may have their own opinions, even if employed at the same unit as another dietitian replying to the survey. As information is self-reported in a survey and not verified, there is a risk of human error. Online studies carry additional risks, including dishonest answers, misunderstanding the question, selecting an answer without fully understanding it, and overall survey fatigue. Lastly, as a singular study, further exploration and research are warranted.

As recognition via engagement and acknowledgment is desired by RDNs, it is apparent that RDNs need to be an integral part of the interdisciplinary NICU rounds team to showcase and validate their expertise and their roles to other providers. Without this, the longevity to continue in the role may be limited or lack advancement to broaden the role. Expanded leadership or research opportunities may increase financial compensation and extend time in the NICU RDN role.

## 5 Conclusion

The survey provides valuable insights for not only managers of dietitians but also professional societies to build programs and retention opportunities to ensure the most vulnerable patients have the best quality of care possible. These results should be beneficial for a myriad of readers who currently engage with or are considering the role of the neonatal dietitian. For organizations that support the role of the neonatal dietitian, the competencies required to be successful in the field should be considered to better prepare those entering the role. Supporting dietitians who wish to pursue infant nutrition specialties would benefit from the ability to have increased options in pediatric internships or neonatal fellowships. For hospitals that employ neonatal dietitians, financial support or reimbursement for continuing education is not consistently an employee benefit, which may play a key role in job satisfaction. Finding innovative ways, such as incorporating artificial intelligence to enhance continuing education, is vital for current RDN practice. Additionally, role advancement opportunities and a compensation ladder should be initiated to encourage the retention of employees, which ultimately provides cost savings. The neonatal dietitian provides the NICU team with the necessary expertise for optimal outcomes in the care of this fragile population, which is vital for patient safety, financial stewardship, and risk management. Lastly, for aspiring neonatal dietitians, gaining insight from tenured NICU RDNs already in the field can help prepare oneself for starting in the role. Learning from their experiences and obtaining guidance on how to succeed and thrive as a neonatal dietitian is invaluable to this specialized career role. The specialized training of the NICU RDN, as a vital team member, will only enhance the value-based care of vulnerable preterm infants. Future studies should consider a cost analysis of the impact of the specially trained, neonatal RDN on infant health outcomes and the length of stay for this fragile, vulnerable population.

## Data Availability

The original contributions presented in the study are included in the article/Supplementary Material, further inquiries can be directed to the corresponding author.
