# Supplementary material for: Elevating the role and professional contributions of the neonatal dietitian for the future: insights from the experts themselves
Source: Front Pediatr. 2025 Aug 26;13:1639283. doi: 10.3389/fped.2025.1639283 (PMC12417191; doi:10.3389/fped.2025.1639283)
Supplement: Supplementary file 1 [file Table1.docx]

| **Supplementary Table S1.**  **Survey Questions** | |  |
| --- | --- | --- |
| **Part I: Employment & Training** | | |
| **Q#** | **Question** | **Response Options** |
| 1 | Are you currently employed as a Neonatal Registered Dietitian Nutritionist? | - Yes (go to Q2) |
|  |  | - No (go to Q1b) |
| 1b | How long ago were you employed as a Neonatal RDN? | a. Less than 1 year |
|  |  | b. 2–4 years |
|  |  | c. 5–10 years |
|  |  | d. More than 10 years ago |
| 1c | What are the reasons you left the role? *(Check all that apply)* | - Moved out of the area |
|  |  | - Poor Job satisfaction |
|  |  | - Non-competitive salary |
|  |  | - Lack of advancement |
|  |  | - Pursued a job outside of dietetics |
|  |  | - Returned to school |
|  |  | - Job with more advancement |
|  |  | - Other (specify) |
| 2 | Are you full or part-time in the NICU? | a. Full-time |
|  |  | b. Part-time |
| 2a | If part-time, how are your hours spent? | a. Adult |
|  |  | b. Pediatrics (in/outpatient) |
|  |  | c. Management |
|  |  | d. Other: ______ |
|  |  | e. Part-time only |
| 3 | NICU Level Classification | a. Level 2 |
|  |  | b. Level 3 |
|  |  | c. Level 4 |
| 4 | Number of NICU Beds | a. 0–10 |
|  |  | b. 10–20 |
|  |  | c. 20–50 |
|  |  | d. 50+ |
| 5 | Institutional support for additional NICU training *(Check all that apply)* | a. Education hours |
|  |  | b. Financial support/reimbursement |
|  |  | c. Conference attendance |
|  |  | d. Shadowing another NICU RDN |
| 6 | How do you obtain NICU education/training? *(Check all that apply)* | a. Conferences |
|  |  | b. Webinars |
|  |  | c. Self-study (books/podcasts) |
|  |  | d. AND-sponsored events |
|  |  | e. Industry-supported events |
|  |  | f. Advanced training (MS, DCN, PhD, etc.) |
|  |  | g. ASPEN-sponsored events |
| 7 | Have you used ChatGPT for learning? | a. Yes |
|  |  | b. No |
| 8 | Does your NICU have 20 beds per FTE RDN? | a. Yes |
|  |  | b. No |
| **Part II: Experience & Recognition** | | |
| **Q#** | **Question** | **Response Options** |
| 1 | When you started as a NICU RD, what experience did you have? *(Check all that apply)* | a. Pediatric clinical experience |
|  |  | b. Prior WIC nutritionist |
|  |  | c. Pediatric intern rotation |
|  |  | d. Pediatric cert/fellowship |
|  |  | e. Adult experience only |
|  |  | f. None |
|  |  | g. Other: ______ |
| 2 | Onboarding training by a tenured NICU RD? | a. Yes |
|  |  | b. No |
| 3 | Do/Did you feel like a valued team member? | a. Yes |
|  |  | b. No |
| 4 | Other credentials valuable to your role? *(Check all that apply)* | a. CSP/CSPCC |
|  |  | b. CNSC |
|  |  | c. CLC |
|  |  | d. IBCLC |
|  |  | e. Master’s degree |
|  |  | f. Doctorate |
| 5 | Accredited education from industry—how did you find it? *(Check all that apply)* | a. Valuable |
|  |  | b. Helpful |
|  |  | c. Coerced |
|  |  | d. Biased |
| 6 | How are/were you recognized in your role? *(Check all that apply)* | a. Noticed when absent |
|  |  | b. Engaged in NICU rounds |
|  |  | c. Thanked by families |
|  |  | d. Admin appreciation |
|  |  | e. Consulted regularly |
|  |  | f. None of the above |
| 7 | Are your notes read by other HCPs? | a. Yes |
|  |  | b. Sometimes |
|  |  | c. Rarely |
|  |  | d. No |
| 7a | If Sometimes/Rarely/No, what are barriers? *(Check all that apply)* | - EMR is cumbersome |
|  |  | - Providers uninterested |
|  |  | - Don’t know if notes are read |
| 8 | Does your job include any of the following? *(Check all that apply)* | a. Rounding in other units |
|  |  | b. NICU Follow-up Clinic |
|  |  | c. Management |
|  |  | d. Research/Grants |
|  |  | e. Teaching residents |
|  |  | f. Precepting interns |
|  |  | g. Milk Lab/Formula Room |
| 9 | Do/Did you feel you have/had the following? *(Check all that apply)* | a. Advancement opportunities |
|  |  | b. Motivation |
|  |  | c. Personal satisfaction |
|  |  | d. Job security |
|  |  | e. Team engagement |
|  |  | f. Fair compensation |
|  |  | g. Appreciation |
| 10 | Do/Did you plan to continue in your role for: | a. Up to 2 years |
|  |  | b. 3–5 years |
|  |  | c. More than 5 years |
| 11 | Employment arrangement? | a. Directly by hospital |
|  |  | b. Contracted (e.g., Sodexo) |
|  |  | c. Other |
